# Innate immunity in hepatitis B and D virus infection: consequences for viral persistence, inflammation, and T cell recognition

**DOI:** 10.1007/s00281-021-00864-x

**Published:** 2021-05-21

**Authors:** Maura Dandri, Antonio Bertoletti, Marc Lütgehetmann

**Affiliations:** 1 I. Department of Internal Medicine, Center for Internal Medicine, University Medical Center Hamburg-Eppendorf, Martinistr. 52, D-20246 Hamburg, Germany; 2 Hamburg-Lübeck-Borstel-Riems partner site, German Center for Infection Research (DZIF), Hamburg, Germany; 3 Singapore Immunology Network (SIgN), Agency of Science Technology and Research (ASTAR), Singapore, Singapore; 4 Program Emerging Infectious Diseases, Duke-NUS Medical School, Singapore, Singapore; 5 Institute of Microbiology, Virology and Hygiene, University Medical Center Hamburg-Eppendorf, Hamburg, Germany

**Keywords:** Hepatitis B virus, Hepatitis D virus, Innate immunity, Hepatocytes

## Abstract

Chronic infections with human hepatitis viruses continue to be a major health burden worldwide. Despite the availability of an effective prophylactic vaccine against the hepatitis B virus (HBV) and of antiviral agents efficiently suppressing HBV replication, more than 250 million people are currently chronically infected with this hepatotropic DNA virus, and resolution of chronic hepatitis B (CHB) is rarely achieved. Moreover, coinfection with the hepatitis D virus (HDV), a human RNA satellite virus requiring the envelope proteins of HBV for productive viral spreading, substantially aggravates the disease course of CHB. The molecular mechanisms by which these viruses interact with each other and with the intrinsic innate responses of the hepatocytes are not fully understood. While HBV appears to avoid innate immune recognition, HDV elicits a strong enhancement of innate responses. Notwithstanding, such induction does not hamper HDV replication but contributes to liver inflammation and pathogenesis. Intriguingly, HDV appears to influence the ability of T cells to recognize infected hepatocytes by boosting antigen presentation. This review focuses on current knowledge regarding how these viruses can shape and counteract the intrinsic innate responses of the hepatocytes, thus affecting the immune system and pathogenesis. Understanding the distinct strategies of persistence that HBV and HDV have evolved is central for advancing the development of curative therapies.

## Introduction

Hepatitis B virus (HBV) remains the major etiological agent of chronic viral hepatitis worldwide. Persistent HBV infection is characterized by various degrees of liver inflammation, which bears the risk, over decades, to develop liver cirrhosis and hepatocellular carcinoma (HCC) [1]. The current death toll is around 880.000 deaths a year [2]. Alarmingly, the number of deaths associated with chronic viral infections is even increasing [3]. Nucleos(t)ide analogs (NUCs) are safe and well-tolerated approved antiviral agents. Because of their ability to suppress HBV replication efficiently and their high barrier to resistance, they have become the gold standard for chronic hepatitis B (CHB) treatment [4]. However, loss of circulating viral antigens (HBeAg, HBsAg) and seroconversion remain rare under NUC treatment, committing most patients to long-term antiviral therapy. Although NUCs have been shown to prevent disease progression in most patients and to reduce the risk of developing HCC in non-cirrhotic patients, NUC discontinuation is often bound to the relapse of viral activity. This is mainly due to the fact that NUCs do not target the HBV genome template that is formed from incoming virions in the nuclei of infected hepatocytes [5]. In contrast, pegylated interferon alpha (peg-IFNα) is the only approved finite treatment for chronic HBV infection, despite its limited capacity to induce seroconversion and association with side effects [6]. Thus, undetectable HBV DNA levels in the serum and HBsAg loss, which are considered endpoints for a functional cure, are rarely achieved with existing treatments [4]. Therefore, the current efforts to advance treatment options for CHB aim to increase off-treatment response rates or functional cure rates [7].

The molecular mechanisms determining either effective HBV recognition and clearance, or persistence and pathogenesis are not fully elucidated. Acute HBV infection is known to resolve spontaneously in approximately 95% of immunocompetent adults, leading to the development of long-lasting immunity [8]. In contrast, 90% of children infected before 1 year of age develop a chronic HBV infection [9]. In general, the resolution of HBV infection requires an effective viral recognition and concerted induction of innate and adaptive immune responses. Both animal and clinical studies demonstrated that in acute self-limited HBV infection, both CD8^+^ T cell and CD4^+^ T cell responses to HBV proteins are strong and polyclonal [10], whereas in chronically infected individuals, immune responses appear weak and narrowly focused [8, 11]. The inability to effectively suppress HBV infection therefore results in the persistence of high quantities of viral antigen over the years. Such chronic presentation of viral antigens progressively suppresses virus-specific T cell immunity, which appears particularly compromised in old CHB patients [12, 13]

Despite the clear role of adaptive immune responses to resolve HBV infection, both unique replication characteristics of this hepatotropic virus and its ability to avoid or even affect intrinsic innate responses appear to be key elements in determining the failure of effective HBV recognition as it is observed in the course of CHB.

The disease course of CHB is substantially aggravated by co- or super-infection with the hepatitis delta virus (HDV), which is the only known satellite virus infecting humans. According to the World Health Organization (WHO), at least 12 million individuals are HBV/HDV co-infected worldwide, although recent metanalyses indicated these numbers may be substantially higher [14]. HDV infection causes the most severe form of chronic viral hepatitis, since it is associated with more rapid progression to cirrhosis, liver decompensation, HCC, and death [15]. As a defective RNA viroid, HDV needs the expression of HBV envelope proteins for the productive release of HDV particles and propagation among human hepatocytes. Because HDV shares the same envelope proteins of HBV, HDV infection can be prevented by hepatitis B immunization in HBV-negative individuals. However, treatment options for patients with chronic HDV (CHD) are limited. Off-label use of pegylated interferon alpha (peg-IFNα) shows limited efficacy, is curative in a minority of patients, and is associated with frequent and sometimes severe side effects [16]. The development of novel in vitro and in vivo systems for HBV and HDV infection has opened new venues for the preclinical assessment of new therapeutic options [17, 18]. As a result, Myrcludex B/bulevirtide (BLV), a synthetic peptide-blocking HBV and HDV cell entry, has now reached the clinic and was conditionally approved for the treatment of HBV/HDV-co-infected patients in Europe and Russia in 2020. In clinical trials, Bulevirtide has shown excellent safety and strong effectiveness in lowering HDV RNA loads [19] both alone and combined with peg-IFNα [20]. Further, promising treatment options include peg-IFN-lambda [21, 22], lonafarnib, which is a farnesyl transferase inhibitor, and nucleic acid polymers [23, 24]. Despite these encouraging therapeutic progresses, the molecular mechanisms responsible for the more severe disease progression observed in CHD compared to chronic HBV mono-infection are not yet fully understood. The strict host and tissue tropism of these viruses have hindered in-depth understanding of the host mechanisms involved in HBV and HDV sensing and the strategies adopted by HBV to escape recognition. Elucidating the interplay between HBV and HDV in infected cells, as well as the strategies used by both viruses to evade and modulate immune responses, remains a key effort for the development of curative therapies.

## HBV replication and persistence strategies

**Fig. 1:**
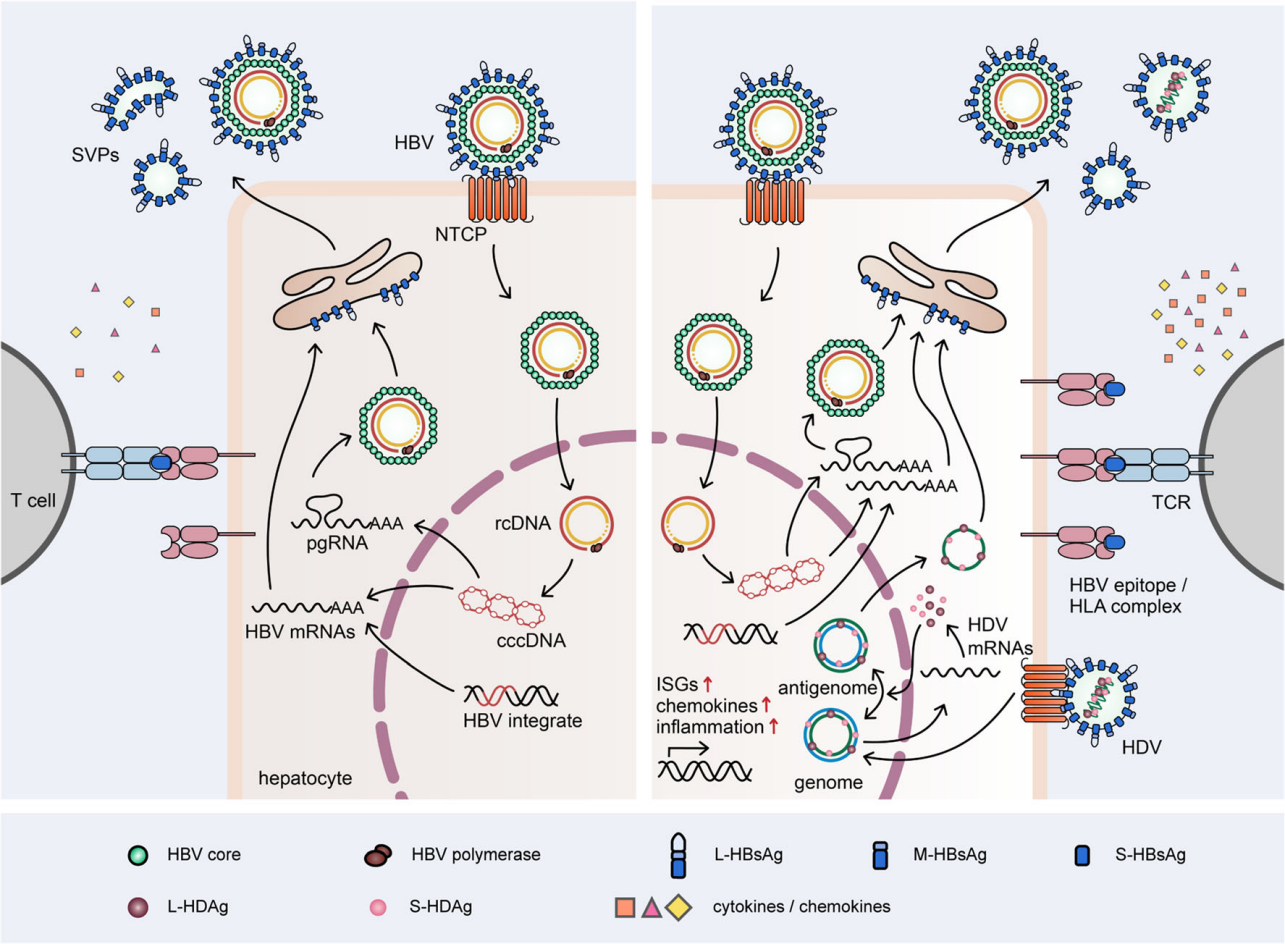
Schematic representation of the replication cycle of HBV in mono-infected hepatocytes and of HBV and HDV in co-infected hepatocytes, pointing out the enhancement of innate genes and increased antigen presentation

The hepatitis B virus is a small blood-borne enveloped DNA virus that can cause both acute and chronic infection by targeting the hepatocytes, which are the only cells susceptible to infection. Typical of HBV is not only its high tissue and species specificity, but also a unique genomic organization and replication mechanism, which involves the formation of an over-length RNA intermediate and the utilization of a reverse transcriptase [25]. The infectious viral particle consists of a spherical lipid envelope containing a single small circular partially double-stranded DNA (rcDNA) molecule of about 3200 nucleotides, which is covalently linked to the viral polymerase and packaged within a nucleocapsid formed by the core protein (HBcAg) [25]. The viral membrane is formed by host-derived lipids and three HBV envelope proteins that are named, according to their size, preS1 (or large), preS2 (or middle), and S (or small). The three HBV envelope proteins share the C-terminal extremity, which corresponds to the S domain of the small protein, while the middle and large proteins display N-terminal extensions of 55 (preS2) and, genotype-dependent, 107 or 118 amino acids (preS1), respectively [26]. The same C-terminal domain bears the region coding for the surface antigen (HBsAg). A crucial step in the entry process of HBV into the human hepatocytes is the high-affinity binding of the N-terminal domain of the preS1 protein to the hepatocyte-specific receptor, the Na^+^-taurocholate cotransporting polypeptide (NTCP) [27, 28]. Notably, the entry of both HBV and HDV is efficiently blocked by a small myristoylated lipopeptide derived from the preS1 domain of the large envelope protein [18, 29–31]. To establish a productive HBV infection, the viral genome needs to be conveyed to the hepatocyte nucleus (Fig. 1), where the key molecule of viral persistence, the covalently closed circular DNA (cccDNA) minichromosome, is built from the rcDNA with the support of distinct, not yet fully characterized, cellular enzymes. The formation of the cccDNA is therefore a multi-step process requiring the participation of the cellular DNA repair machinery and its association with histone and non-histone proteins (for a review, see [32]). The cccDNA serves as a template for the production of all viral RNAs that are transcribed from four largely overlapping main open reading frames. Distinct promoters and enhancer regions regulate the transcription of both subgenomic RNAs, like those responsible for the production of the envelope proteins and an over-length pregenomic RNA (pgRNA). The transcription of the pgRNA is not only indispensable for viral replication, but is also responsible for the translation of the polymerase and of the core proteins. Through the reverse transcription of the pgRNA within the nucleocapsids, newly formed rcDNA-containing nucleocapsids are enveloped and secreted as progeny viruses via the endosomal secretory pathway [33].

The smallest subgenomic RNA produces the regulatory X protein (HBx), which was reported to interfere with several cellular pathways and transcription factors and to be recruited onto the cccDNA [32, 34]. Moreover, a major function of HBx that has emerged in the last years regards its ability to hinder the host’s attempts to silence cccDNA transcription [35–37]. By binding to the damaged DNA binding protein 1 (DDB1) [38], HBx triggers the degradation of the structural maintenance of chromosomes 5/6 complex (SMC5/6), a multi-functional DNA-binding complex involved in chromosome dynamics and stability [39]. Thus, HBx expression appears fundamental to counteract the SMC5/6 host restriction factor and maintain active transcription of the cccDNA minichromosome. Therapeutic strategies aiming at abrogating HBx production, such as siRNA technologies, were recently shown to enable reappearance of the SMC5/6 complex and silencing of the cccDNA minichromosome in HBV-infected chimeric mice [40].

Infected hepatocytes also secrete the non-structural pre-core protein, the so-called E antigen (HBeAg), as well as high amounts of subviral particles (SVPs), which are mainly composed of envelope proteins (HBsAg) but lack the capsid and the viral genome. High levels of circulating viral antigens are thought to contribute to establishing HBV persistence and a state of immune tolerance [41]. Of note, HBV DNA sequences are also found integrated into the host genome in the liver of infected individuals [42, 43]. Although HBV DNA sequences are often truncated and highly rearranged, they can contribute to the production of viral proteins, in particular, HBsAg. Moreover, additional virological markers like hepatitis B core-related antigens (HBcrAg) and enveloped particles containing HBV RNAs or empty capsids are also secreted from HBV replicating cells into the bloodstream [33]. Their relationship with cccDNA transcription, their clinical potential as surrogate biomarkers [44], and their role in the HBV life cycle, immunomodulation, and pathogenesis still need to be elucidated.

The persistence of the HBV minichromosome in the liver of CHB patients is considered the main virological reason for the rebound of HBV commonly determined in serum after cessation of NUC therapy. In an environment where liver inflammation is controlled and hepatocyte turnover remains low, the intrahepatic cccDNA pool appears stable [45]. However, knowledge of the half-life of individual cccDNA molecules is still limited, with reports estimating half-lives spanning from months to years [46–48]. To note, an accurate definition of the longevity of individual cccDNA molecules remains technically challenging. Adding complexity, the occurrence of new infection events is thought to play a key role in determining maintenance and renewal of intrahepatic cccDNA loads even under NUC therapy, due to the inability of polymerase inhibitors to achieve complete suppression of HBV replication [46].

It is conceivable that the kinetics of cccDNA decay are substantially affected by various biochemical and immunological conditions. Without a doubt, immune cells have the ability to recognize and destroy the cccDNA together with the infected hepatocytes, events that are expected to lower the intrahepatic cccDNA levels. Furthermore, the immune-mediated elimination of infected cells is bound to promote compensatory hepatocyte proliferation. Cell division has been shown not only to dilute the existing cccDNA molecules among dividing cells, but also to facilitate their destabilization and loss, thus leading to a substantial reduction of the intrahepatic cccDNA pool [49].

Studies with HBV-related viruses have shown that a pool of cccDNA molecules can be established not only from rcDNA molecules infecting the cells via the NTCP receptor entry pathway “external route,” but also through an “internal route” redirecting newly synthesized rcDNAs into the nucleus instead of promoting cell egress [49, 50]. However, the efficacy of such an “internal route” in HBV-infected human hepatocytes has been recently questioned, since both in vitro [43] and in vivo [31, 49] studies provide accumulating evidence that amplification and replenishment of the HBV cccDNA pool are mainly supported by new infection events. Understanding cccDNA biology and whether the transport of newly synthesized rcDNA molecules into the nucleus of human hepatocytes are key events determining maintenance of the cccDNA pool remain mandatory to assist the design of future therapeutic interventions. In this regard, the development of therapeutic approaches able to lower cccDNA amounts, to trigger its silencing, and to guard the cells from new infection events represent the main goals for achieving a functional cure. Together with strategies promoting restoration of the HBV-specific antiviral immune responses, a complete HBV cure could even be envisioned [5, 7, 24].

## HDV replication and persistence strategies

The viral genome of HDV is a circular, single-stranded, negative-sense (−) RNA of approximately 1680 nucleotides. In the nucleus of infected human hepatocytes, the viral genome appears as a rod-like structure with broad intramolecular base pairing. This leads to the accumulation of three distinct RNA forms: the genomic RNA (−); the antigenomic RNA (+), which is an exact complement of the genomic RNA; and a smaller linear mRNA (+) encoding two isoforms of the only viral protein, the hepatitis delta antigen (HDAg). The small (S-) HDAg (24 kDa, 195 amino acids) is important for virus replication, while the large (L) variant (27 kDa, 214 amino acids), which is generated by an RNA editing event induced by the cellular enzyme adenosine deaminase acting on RNA (ADAR), is essential for virus assembly [51]. Abrogation of the stop-codon within the HDV antigenome enables the extension of the mRNA open reading frame and translation of L-HDAg, which harbors a nuclear export signal. The host RNA polymerases drive HDV replication using the antigenome as a template and are also responsible for the transcription of the HDV mRNA (Fig. 1). Using a double rolling-circle amplification process, HDV genomes and antigenomes are first generated as oligomers that get self-cleaved into RNA monomers through their intrinsic ribozyme activity. Newly formed HDV-RNA is associated with L-HDAg and S-HDAg to generate a ribonucleoprotein (RNP) complex. This complex is enveloped through budding into an ER-derived lipid bilayer carrying the three HBV envelope proteins to generate new virions [52].

The balance between genomic and antigenomic RNA appears crucial in guaranteeing persistence of HDV replication and is highly regulated by the two forms of HDAg, as well as through epigenetic modifications [53, 54]. Post-translational modifications are also known to play a key role in HDV replication and morphogenesis. Notably, farnesylation of the L-HDAg is essential for enabling the interaction of the HDV RNP complex with the HBsAg in the cytoplasm, thus favoring the assembly of HDV virions [55].

HBV plays an essential role as a helper virus for HDV transmission. However, HDV infection was shown to persist in patients for years also in the presence of very low levels of HBV infection [56, 57]. Intriguingly, HDAg-positive cells have been detected after liver transplantation for up to one and half years in the absence of HBV replication [58, 59], and infection studies in human liver chimeric mice revealed that HDV can infect and persist in vivo for at least 6 weeks in the absence of HBV [60]. Moreover, these in vivo experiments demonstrated that HDV mono-infection could be converted into a productive infection by super-infection with HBV [60], thus highlighting the endurance capacity of HDV in quiescent human hepatocytes. Of note, recent in vitro and in vivo studies revealed that HDV may even be rescued by alternative enveloped viruses, such as flaviviruses, hepaciviruses, and vesiculoviruses [61]. Particularly intriguing was the observation that HDV could be propagated by HCV in the liver of co-infected humanized mice, thus providing experimental evidence that HBV envelope proteins are not strictly required for HDV cell egress. However, clinical analysis of chronically infected HCV patient cohorts failed to document cases of HDV/HCV co-infection without HBV, thus indicating that HDV propagation mediated by HCV infection may rarely occur in a real clinical setting [62–64]. Since the interaction between the farnesylated L-HDAg and the HBsAg is weak, the evolutionary advantage of using HBV as a helper virus to exit the hepatocytes is not fully elucidated. Yet, it is conceivable that not only the tolerogenic liver environment and the peculiar low immune recognition profile of HBV (see below), but also the high production of HBV envelope proteins and of SVPs, which may serve as immunological decoy also for HDV [65], represent key benefits for HDV/HBV co-replication.

Keeping in mind the requirement of HBV envelope proteins for HDV cell-to-cell propagation, it could be hypothesized that immune-mediated cell turnover in HBV/HDV chronically infected livers could not only accelerate cccDNA loss but also affect HDV persistence. However, in vitro and in vivo experiments revealed that cell division promoted the clonal expansion of HDV-positive cell clusters, thus enabling HDV to propagate and replicate among dividing human hepatocytes even in the absence of HBV [66]. These findings highlight the strong persistence capacities of this unique RNA virus in the liver of HBV/HDV chronically infected patients.

## HBV and HDV recognition by intrinsic innate mechanisms of the hepatocytes

The ability of the innate immunity to recognize intracellular pathogens, like viruses, is central to initiate the first line of defense and to coordinate immune responses adequate to achieve the control of the infection. A large range of pathogens is sensed through germline-encoded pattern recognition receptors (PRRs) that are present either on the cell surface or within multiple intracellular compartments. These PRRs include membrane-bound Toll-like receptors (TLRs); cytosolic DNA sensors, such as members of the AIM2 family; and numerous cytosolic RNA sensors, like the RIG I like receptors (RLRs) with their main players: the retinoic acid-inducible gene I (RIG-I) and melanoma differentiation-associated gene 5 (MDA5). These receptors are specialized to recognize unusual structures like viral proteins or nucleic acids [67]. Their activation initiates the recruitment of distinct sets of adaptor molecules, such as Myd88 (myeloid differentiation primary response gene 88), MAVS (mitochondrial antiviral-signaling protein), STING (stimulator of interferon genes), IFI16, and TRIF (TIR-domain-containing adapter-inducing interferon-β), which trigger the main signaling pathways of NF-kB and interferon regulatory factors (IRFs). Nuclear translocation of these factors culminates in the induction of interferon-stimulated genes (ISGs) and production of different inflammatory cytokines, interferons (type I/III IFNs), and chemokines (reviewed in [68]).

Primary human hepatocytes (PHHs) express a broad range of PRRs [69] and have the ability to sense various pathogens, as shown by studies with the hepatitis C virus (HCV). In this regard, both experimental studies [70–73] and analyses of liver specimens from HCV-infected patients provided clear evidence that HCV induces the upregulation of ISGs and a strong interferon response [74–76]. The enhancement of various human ISGs and chemokines, like TGFβ1 and IP10, was also demonstrated in the absence of the adaptive immune system, in HCV-infected human hepatocytes within the liver of immunodeficient uPA chimeric mice [77]. Of note, HCV is an RNA virus whose replication cycle exclusively takes place in the cytoplasm of infected cells. It is therefore plausible that PRRs, like RIG-I, MAVS, and TLR3, can sense cytosolic HCV RNA despite the ability of HCV proteins to attenuate the IFN response by counteracting components of the innate immune signaling [68]. Nevertheless, type I and III interferons are produced during HCV infection, and interferon-based treatments suppress HCV replication both in vitro [78] and in vivo [77, 79], even though sustained ISG expression has been associated with weaker responses to IFN-based treatment [80].

In contrast to HCV, after entering the hepatocytes, the HBV DNA is transported to the cell nucleus" instead of "into" - since the genome may get released from the nucleocapsid at the level of the nuclear membrane. Above all, the mimicry ability of the HBV DNA genome to reside as a minichromosome in hepatocyte nuclei appears to be a key replication strategy of this virus to avoid innate immune recognition. Transcribed HBV RNAs are generated from the cccDNA by cellular enzymes and resemble host messenger RNAs with their 5′ cap and 3′poly(A) tail, thus offering poor opportunities to ignite recognition. Only the HBV pgRNA contains an unusual hairpin loop as packaging signal [81] and was shown to induce cytosolic PRRs (RIG-I) in certain experimental conditions [82]. Nevertheless, the pgRNA becomes rapidly encapsidated by the core proteins in the cytoplasm, thus protecting the HBV replicative intermediate from PRR recognition. Such ability of HBV to escape a strong induction of the so-called antiviral state led researchers to stamp HBV as a “stealth” virus [73].

Both studies in chimpanzees [83] and in patients with acute infection [84] showed that HBV neither triggers the induction of type I/III interferons nor clearly enhances ISGs, indicating that either the sensory pathways are unable to recognize HBV or that HBV can actively block these pathways. Likewise, in vitro studies with HBV-infected human hepatocytes failed to detect upregulation of ISGs [72], and liver specimens from patients with CHB were reported to express ISG levels similar to those obtained from control individuals [85]. Nevertheless, this study showed that innate responses could be activated upon ex vivo incubation of fresh liver biopsies with TLR3 agonists.

Human liver chimeric mice are based on the repopulation of the mouse liver with primary human hepatocytes. Because these mice lack NK cells and functional adaptive immune responses, the model offers the opportunity to dissect interactions occurring between human hepatotropic viruses and intrinsic innate responses of the human hepatocytes in vivo. We and others employed these systems to investigate the capacity of human hepatocytes to sense different hepatitis viruses in vivo [21, 86], as well as the antiviral effects of therapeutic cytokines like interferons [22, 40, 87, 88] and of HBV-specific immune cells [89–91]. These studies revealed that HBV induces a much weaker and barely detectable enhancement of innate immunity genes [86, 92] in comparison with infections with HCV [77] or HDV [86].

As an RNA virus, HDV is expected to be recognized by various PRRs, and in particular RNA sensors like RLRs, MDA5 and to activate downstream signaling proteins (i.e., MAVS) and transcription factors, which can translocate into the cell nucleus and initiate the transcription of IFN and ISG genes. HBV/HDV co-infection in humanized mice was shown to provoke a sustained induction of the antiviral state of the human hepatocytes by promoting the enhancement of classical human ISGs, genes involved in antigen presentation (see below), and the induction of inflammatory and pro-fibrogenic cytokines (i.e., IP10, IFN-β, TGF-β), both at the transcriptional and protein levels [86]. In this regard, TNFα was recently shown to play a key role in HDV-mediated liver inflammation [93]. A similar HDV-mediated induction of ISGs was also observed in vitro [94] and in other mouse models [95–97]. The increased amounts of ISGs and cytokine levels may be a key determinant of liver inflammation in HDV infection, thus explaining the more severe course of disease observed in CHD patients. The enhancement and nuclear translocation of STAT in human hepatocytes also indicated that HDV triggered the JAK/STAT signaling cascade in HBV/HDV co-infected livers. Notably and in line with previous in vitro studies [98], nuclear accumulation of STAT proteins appeared most pronounced in cells displaying lower HDAg levels, suggesting that interference mechanisms may be active in the presence of high HDV protein levels [86]. Microarray analyses also reported the activation of a broad range of ISGs and production of IFN-β and IFN-λ in hepatoma cell lines (HepG2-NTCP cells) and PHH cultures infected with HDV [99]. Of note, HDV-mediated induction of IFN appeared strongly reduced upon depletion of MDA5, thus indicating that this innate cellular component can act as a key sensor of HDV RNA recognition [96, 100]. However, it remains unknown whether MDA5 can be also transported to the nucleus, which is the replication site of HDV. Thus, the ability of this virus to replicate in a “protected” cellular compartment may be seen as a countermeasure to avoid recognition from PRRs or other cellular restriction mechanisms as it has been recently described for HBV (i.e., SMC5/6 complex).

**Fig. 2:**
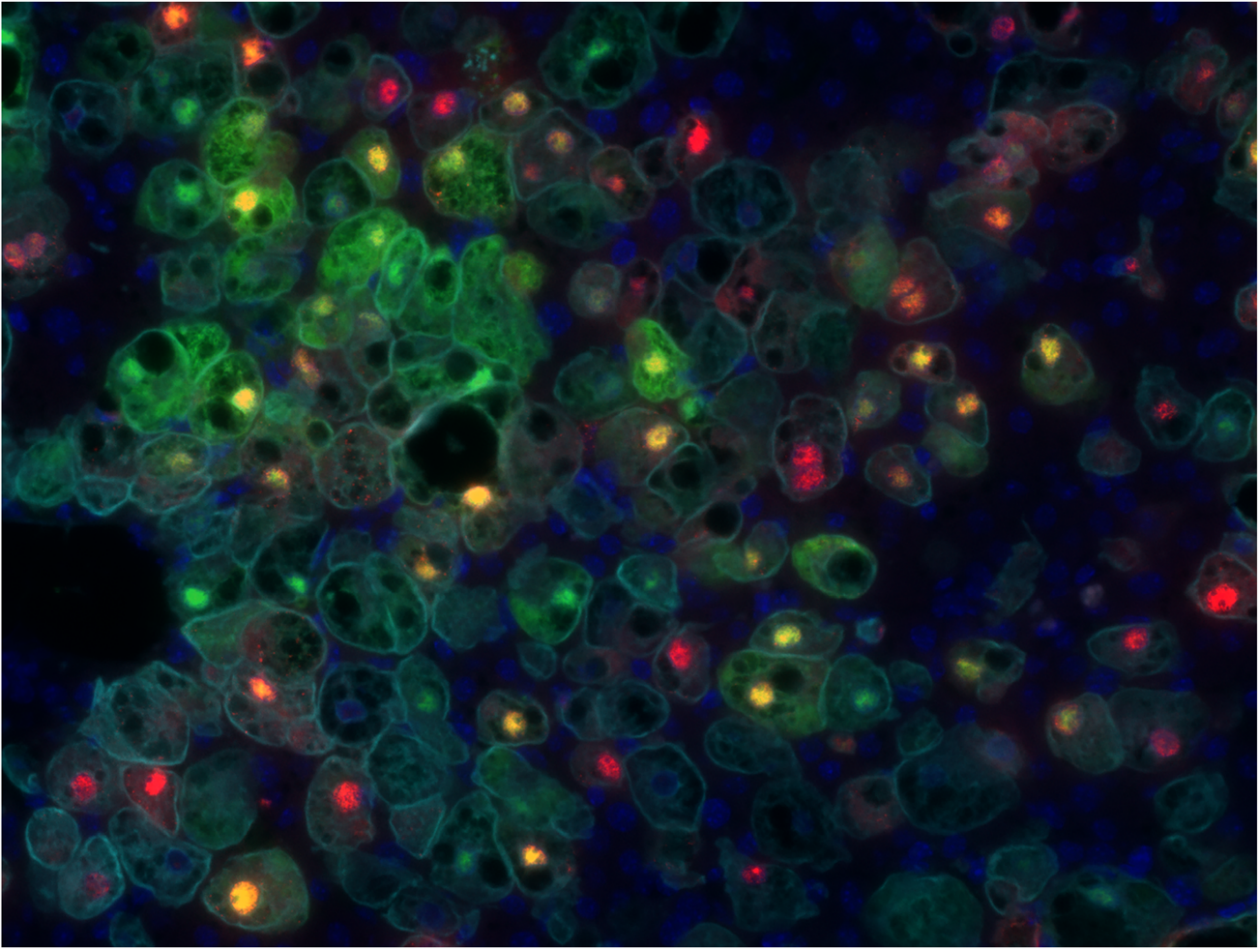
Immunofluorescence staining of cryostat sections of a HBV/HDV-infected humanized mouse liver showing primary human hepatocytes (anti-CK18, 1:400, Dako, Denmark) expressing viral markers of both viruses within the same nuclei (merged yellow signal), as well as the presence of cells producing either high levels of HBcAg (green; rabbit anti-HBcAg, 1:2000, Dako) or nearly only HDAg (red signal; anti-HDAg-positive human serum, 1:8000), indicating strongly different and possibly fluctuating levels of HBV and HDV infection coexisting in immune-deficient chronic infected livers. The nuclei were stained by Hoechst 33258 (1:20,000 diluted, Invitrogen). Stained sections were mounted with fluorescent mounting media (Dako), analyzed with a fluorescence microscope BZ8710 (Keyence, Osaka, Japan), and photographed using a ×40 magnifying lens

It is noteworthy that different levels of HBV and HDV infection can coexist among human cells within the same liver even in immune-deficient liver chimeric mice. As shown in Fig. 2, human hepatocytes expressing almost exclusively high levels of HDAg can be observed near cells expressing both viruses, as well as cells positive only for HBV markers. Moreover, the development of HBV viremia and the increase of intrahepatic cccDNA loads appeared substantially delayed in HBV/HDV co-infected mice in comparison with HBV mono-infected animals, suggesting that the stronger antiviral state exerted by HDV during viral spreading could interfere with HBV replication and establishment of new infection events [18, 101]. Such moderate HDV-mediated suppression of HBV activity could occur also in the absence of the adaptive immune system, as it is the case in chimeric mice, thus supporting the notion that intrinsic innate mechanisms elicited within the hepatocytes and/or direct virus/virus interferences account for a certain dampening of HBV productivity. Although the exact molecular mechanisms by which HDV can suppress HBV remain to be elucidated, these observations are in agreement with studies reporting lower levels of HBV infection in HBV/HDV co-infected patients [56].

Single-cell analyses based on in situ RNA hybridization showed that the activation of innate genes is clearly detectable not only in HBV/HDV co-infected livers [86, 96, 99], but also within the human hepatocytes [21]. While some studies reported induction of ISGs and IFN also in the setting of HDV mono-infection [95–97], a clear induction of the same intrinsic immune responses could not be detected in the setting of HDV mono-infection in immune-deficient humanized mice [21]. The mechanisms underlying such differences are unclear. The low amounts of infected cells achievable in vivo in the setting of HDV mono-infection may in part impact the type and amplitude of intrahepatic HDV sensing. Of note, extracellular vesicles released from HDV mono-infected cells have been recently shown to bear the ability to induce pro-inflammatory cytokines in macrophages and peripheral blood mononuclear cells [102], thus indicating that different mechanisms may participate in intercellular HDV sensing. Moreover, one could speculate that the co-existence of both viruses enabling ongoing HDV spreading among hepatocytes may play a determinant role in promoting the enhancement of most ISGs, as well as the production of pro-inflammatory cytokines. If this is true, abrogation of new infection events would be expected to lower liver inflammation in CHD infection—and provide at least in part a rationale for the amelioration of most biochemical parameters as it has been observed in the clinical trials investigating the efficacy of the HBV/HDV entry inhibitor bulevirtide [103].

Altogether, the abovementioned studies provide strong evidence that the presence of HBV in human hepatocytes co-infected with either HCV or HDV [72, 86] did not prevent the enhancement of intrinsic innate responses promoted by these RNA viruses. Therefore, such studies support the hypothesis that HBV remains nearly invisible to PRRs within the infected hepatocytes. While the ability of HBV to escape innate recognition in infected cells encounters large consensus in the scientific community, it remains unclear whether HBV can actively suppress or hinder the induction of innate mechanisms in infected cells at least in certain conditions. In this respect, downregulation of some innate genes was observed in liver samples from CHB patients [104], while other studies reported the ability of the precore/HBeAg to target and hinder the induction of the TLR signaling pathways, thus supporting the notion that HBV proteins may actively contribute to the evasion of the innate responses [105]. Moreover, the HBx protein and the HBV polymerase were reported to block the induction of innate immunity genes in vitro [106, 107]. However, the use of different experimental models and viral protein expression levels may in part explain the controversial data reported. Yet, persistent production of HBsAg and HBeAg have shown to exert immune-modulating functions contributing to viral persistence [8, 105, 108–110] and to the B cell exhaustion detected in chronically HBV-infected individuals [111–115].

It can be noted that also HDV adopts unusual replication modalities, which enable the virus to limit contacts with the intracellular PPRs by nuclear compartmentalization of the HDV RNA-RNA replication. In addition, the formation of a circular RNA genome without accessible 5′ or 3′ ends prevents PRR recognition by RIG I [116], while other viral RNA motives (like GC rich elements) that are potentially recognized by innate defense mechanisms are shielded by the delta antigens forming tight ribonucleoprotein (RNP) complexes [117] that may remain spatially inaccessible to cellular factors such as nucleases. Although it is currently unclear whether the HDAg can contribute or counteract innate immune recognition of HDV infection, the RNP complex is considered to play a key role also in preventing over-accumulation of editing events by ADAR1 at sites of the viral RNA other than the amber/w site. Such control of editing levels appears central to control HDV RNA replication levels and the likely outcome of infection [118].

Similarly to HBV, the HDV RNP complex is further enveloped by HBsAg before budding into the endosomal compartment (for more details on RNA receptors see review [119]), thus limiting the steps where HDV can be recognized by the intrinsic immune mechanisms of the hepatocytes. Finally, HDAg was predicted to generate only few peptides that can be processed and bind to human leukocyte antigen (HLA) molecules; steps that are essential to activate HDV-specific CD8 T cells [120]. Moreover, immune evasion mutations on predicted T cell epitopes were observed in chronic HBV/HDV infected individuals [121].

## Hepatocyte antigen presentation of HBV and HDV and immunogenic properties of HDV

The liver represents a particularly attractive organ for many pathogens. Being exposed to high amounts of food-derived metabolites, toxins, and bacterial products coming from the gut, the liver is characterized by unique tolerogenic properties, where immune activation is kept tightly controlled. While the liver parenchymal cells, the hepatocytes, ensure metabolic and detoxification functions, large amounts of infiltrating and resident immune cells are found in the healthy liver. Innate lymphoid cells, like the Kupffer cells, which are the liver resident macrophages, build together with liver sinusoidal endothelial cells (LSECs) and hepatic stellate cells (HSCs) the so-called non-parenchymal cell (NPC) compartment. In addition to many liver-specific NPCs, natural killer cells and various circulating monocytes, like dendritic cells and lymphocytes, including mucosa-associated invariant T (MAIT) cells, infiltrate the liver and contribute to the orchestration of the immune response [67]. Despite such unique richness of resident and circulating lymphoid and professional antigen-presenting cells, the distinct ability of infected and innate cells to sense and signal the presence of pathogens may differently impact the effectiveness of the immune responses elicited.

Antigen presentation is certainly a required checkpoint to initiate adaptive, pathogen-specific, immune responses. The hepatotropic nature of HBV and HDV and the fact that hepatocytes are in direct contact with blood flow means that naive and effector/memory T cells are in direct contact with the virus-infected parenchymal cells [122, 123]. Priming of naive T cells by antigens exclusively presented by hepatocytes is known to generate T cell tolerance [124], but the mechanisms of antigen presentation during natural infection in the presence or absence of inflammatory events can profoundly alter T cell priming [125] and the ability of effector T cells to recognize infected hepatocytes [126]*.* Recent work in animal models has elegantly demonstrated that priming by HBV-infected hepatocytes causes the induction of HBV-specific CD8^+^ T cells that do not become classical effector CD8^+^ T cells [125]. Moreover, this study pointed out the key role of IL-2 in promoting the expansion and proliferation of primed HBV-specific T cells. Similarly to what is observed in so-called immunotolerant patients (HBeAg+ chronic infection), this specific tolerogenic priming was shown to occur in a liver environment without inflammatory events. Indeed, HBV-specific T cells of immune tolerant CHB patients were shown to expand and become functional upon the addition of IL-2. In contrast, priming in an inflammatory liver environment appears to be mediated mainly by professional antigen-presenting cells (Kupffer cells, endothelial cells). However, such priming was shown to induce more classical effector T cells that became exhausted and could be rescued by anti-PD-1 treatment [125]. Chronic HDV infection was shown to be associated with increased levels of pro-inflammatory cytokines like IL-12 and IL-18 and to engage MAIT cells, which are a subset of innate-like T cells, causing their functional impairment and progressive depletion as the HDV-associated liver disease progresses (Dias 2019). Of note, only a modest MAIT cells decrease was observed in HBV mono-infected patients (Dias), thus suggesting that higher cytokine levels often determined in HDV infection, may contribute to liver damage and disease progression also by the activation and subsequent loss or exhaustion of different innate and adaptive immune responses.

The infection of HDV and its ability to directly activate innate immunity in hepatocytes radically changes the liver microenvironment and the ability of T cells to recognize HBV-infected hepatocytes. Recent work performed in an in vitro HDV infection system, liver biopsies of HBV/HDV co-infected patients and mice with humanized livers, have shown that HDV infection not only enhances the gene expression of HLA class I molecules, *B2M*, immunoproteasome, and co-stimulatory molecules genes, but it also increases the presentation of viral epitopes and, as a consequence, the efficiency of T cell recognition of infected hepatocytes [126].

We do not have data related to how HBV/HDV co-infected hepatocytes might differentially prime naive T cells. However, HDV has clear effects on the presentation of viral antigens to effector CD8^+^ T cells. This could explain the high incidence of CD8^+^ T cell escape mutations found in HDV epitopes [121, 127], but also explain the epidemiological evidence of a better HBV control, or at least frequent HBV suppression, in HBV/HDV co-infected patients than in HBV mono-infected patients. The compact nature of the HBV DNA genome poses a limit on the generation of mutated HBV viruses, and this could in part explain why dually infected HBV/HDV patients become HBsAg-negative at a higher rate than HBV-mono-infected patients [128].

The increased ability of HDV to boost the presentation of HBV antigens was shown to not be exclusive to the co-infected HBV/HDV hepatocytes but also to neighboring HBV mono-infected cells, through a mechanism likely mediated by the increased production of IFN-β and IFN-λ [126]. In this regard, infected or damaged cells and cellular events eliciting pathogen recognition result in the production of a variety of soluble factors, such as danger signals, pro-inflammatory cytokines and chemokines, and extracellular vesicles [102]. These signals may play a pivotal role in promoting bystander innate immune activation of neighboring cells.

The increased antigen presentation induced by HDV also supports the concept that therapeutic interventions designed to boost HBV-specific CD8^+^ T cell responses, with anti-PD1 therapy or with the use of chimeric antibody receptor engineered (CAR) or T cell receptor (TCR)-redirected T cells or TCR-like antibodies might be better suited for the treatment of HBV-HDV chronic hepatitis. The demonstration that HDV can persist intracellularly in replicating human hepatocytes despite blocking re-infection by administering the entry inhibitor bulevirtide [66] suggests that immune-mediated destruction of a substantial fraction of HDV-infected cells is required to substantially lower intrahepatic HDV infection.

## Sensitivity of HBV and HDV to therapeutic cytokines

The persistence of high antigen levels is considered a major factor driving functional exhaustion of HBV-specific immune cells, and various studies indicated a poor restoration of immune cell functions in the early phases of IFN treatment [8, 129–131]. Moreover, the limited rates of cccDNA reduction determined in patients receiving IFN-based therapy [132, 133] do not fully explain the early kinetics of HBsAg decline. Thus, the responsiveness of the hepatocytes to IFN therapy may be central to trigger the initial reduction of viral antigens and thereby facilitate the functional reconstitution of antiviral T cell responses. An early HBsAg decline is indeed observed in patients responding to peg-IFNα therapy [131]. Thus, IFN-based therapy may be beneficial in some patients not only by acting as an immune-modulator, but also by directly lowering HBV RNA levels in infected hepatocytes. Studies in humanized mice showed that administration of conventional IFNα led only to a transient epigenetic suppression of the cccDNA [88] and that the responsiveness of HBV-infected human hepatocytes appeared in part impaired [92]. Moreover, some human ISGs appeared less efficiently induced after one single injection of peg-IFNα in HBV-infected livers compared to the enhancement induced in uninfected animals, suggesting the existence of an initial, albeit partial, impairment of the responsiveness of the HBV-infected hepatocytes to IFNα, which, however, could be breached by repeated administrations of the longer-active peg-IFNα [87]. At any rate, several weeks of treatment with peg-IFNα were sufficient to provoke a strong decrease of circulating and intrahepatic viral antigens despite the absence of immune cell responses in this system [40, 87]. Of note, recent studies showed that not only administration of peg-IFNα but also RNA interference strategies targeting all HBV transcripts could abrogate the production of all HBV proteins, including HBx, in a substantial amount of hepatocytes in vivo, leading to the reappearance of the SMC5/6 complex and cccDNA silencing [40]. Intriguingly, sustained silencing of cccDNA transcription could be maintained in a substantial fraction of infected cells by applying the entry inhibitor bulevirtide, thus by shielding the hepatocytes from new infection events [40].

Due to its narrow genome size and the lack of expression of its own viral polymerase, therapy options for HDV are still limited with peg-IFNα being the drug of common use [23] and more recently bulevirtide, which does not directly block viral activity but rather promotes the reduction of HDV loads by blocking cell entry [103]. Unfortunately, outcomes in patients treated with peg-IFNα remain unsatisfactory and the mechanisms by which IFN exerts anti-HDV effects in human hepatocytes are not yet elucidated. Previous in vitro studies mostly relied on a particular cell culture-derived strain of HDV genotype 1 [134] that resulted insensitive to IFNα in vitro [99]. Contrarily, a patient-derived HDV-1 strain was shown to respond both to peg-IFNα and to peg-IFNλ in human liver chimeric mice [22], thus providing evidence that these therapeutic cytokines can lower HDV loads in infected human hepatocytes also in a system lacking adaptive immune responses. Understanding the mode of action of interferon in HDV-infected cells, as well as the mechanisms responsible for the different responsiveness to interferon among distinct HDV isolates shall greatly assist the design of therapies aiming to accelerate HDV loss and achieve HDV cure.

## Conclusions

Elucidation of the different mechanisms that infected hepatocytes use to unveil the presence of these human hepatotropic viruses to uninfected bystander cells and to different types of resident and circulating immune cells is central to understand key mechanisms determining the resolution of HBV and HDV infection versus persistence. Whereas experimental infection systems and patient analyses support the notion that HBV avoids innate immune recognition, co-infection with HDV appears to cause profound changes in the infected liver. The clear enhancement of various ISGs, the higher production of chemokines and inflammatory cytokines, as well as the increased antigen presentation capabilities determined in HBV/HDV infection may act however as a double sword, boosting the ability of immune cells to recognize infected cells on the one side, but also augmenting liver inflammation and thus accelerating pathogenesis. Through the development of various in vitro and in vivo infection models, as well as of sophisticated technologies enabling the dissection of events occurring at the single-cell level, the role of distinct HBV and HDV proteins in modulating the antiviral responses in infected hepatocytes is gaining recognition, also highlighting the importance of viral activity in counteracting the first line of host defenses. Understanding the interplay between viral proteins and the innate responses remains central for developing curative treatment strategies against HBV and HDV.
